# A curated resource of chemolithoautotrophic genomes and marker genes for CO₂ fixation pathway prediction

**DOI:** 10.1038/s41597-026-06655-z

**Published:** 2026-02-11

**Authors:** Shuichi Kawashima, Yoko Okabeppu, Seiha Miyazawa, Natsuko Ichikawa, Hikaru Nagazumi, Yutaka Nishihara, Takeru Nakazato, Susumu Goto, Ken Kurokawa, Masaharu Ishii, Hiroshi Mori

**Affiliations:** 1 Database Center for Life Science, Joint Support-Center for Data Science Research, Research Organization of Information and Systems, Kashiwa, Chiba 277-0871 Japan; 2 OKBP Inc., Yokohama, Japan; 3 Biological Resource Center, National Institute of Technology and Evaluation, Shibuya-ku, Tokyo 151-0066 Japan; 4 Department of Computer Science and Engineering, Waseda University, Shinjuku-ku, Tokyo 169-8555 Japan; 5 Department of Informatics, National Institute of Genetics, Mishima, Shizuoka 411-8540 Japan

**Keywords:** Applied microbiology, Sequence annotation, Bacteriology

## Abstract

Autotrophic CO₂ fixation is a fundamental metabolic process that enables microorganisms to inhabit carbon-limited environments. Multiple pathways mediate this process, with variants distributed across diverse taxa and some genes shared among pathways, making their identification from genomic data challenging. Here, we present a curated resource comprising pathway-specific KEGG Orthology marker genes and a lightweight, rule-based tool AutoFixMark for predicting the presence of seven known CO₂ fixation pathways in microbial genomes. To support marker gene identification and benchmarking, we compiled two reference datasets: (i) 347 manually curated chemolithoautotrophic genomes from 16 phyla, and (ii) a set of 15 well-characterized chemolithoautotrophic genomes used for defining pathway-specific marker genes. Using these marker genes, we developed AutoFixMark and evaluated its performance against two existing tools, METABOLIC and gapseq. Benchmarking results show that AutoFixMark achieves high precision and recall, particularly for pathways that are underrepresented in current tools. All curated gene sets, prediction rules, the AutoFixMark program, and benchmark datasets are publicly available, providing valuable resources for assessing autotrophic carbon fixation potential in microbial genomes.

## Background & Summary

Understanding carbon fixation in microbes is essential for elucidating global carbon cycling, developing sustainable biotechnological applications, and interpreting microbial contributions to diverse ecosystems. Autotrophic microorganisms employ diverse biochemical strategies to fix carbon. To date, seven distinct natural CO₂ fixation pathways have been characterized: the Calvin–Benson–Bassham (CBB) cycle, the reductive tricarboxylic acid (rTCA) cycle, the Wood–Ljungdahl (WL) pathway, the 3-hydroxypropionate (3HP) bicycle, the 3-hydroxypropionate/4-hydroxybutyrate (3HP/4HB) cycle, the dicarboxylate/4-hydroxybutyrate (DC/4HB) cycle, and the reductive glycine (rGly) pathway^1^. While the taxonomic distribution of these pathways has been summarized at higher ranks (e.g., phylum or class)^2^, a systematic assessment of their presence at the strain level remains largely unexplored. These CO₂ fixation pathways are diverse, with pathway variants occurring in different taxa and certain genes shared across multiple pathways^2^. Consequently, distinguishing these pathways based on gene content alone is often difficult, underscoring the need for dedicated tools capable of inferring CO₂ fixation pathways directly from genomic data. Existing tools such as METABOLIC and gapseq offer general metabolic pathway predictions^3,4^, yet their accuracy in detecting specific CO₂ fixation pathways has not been comprehensively evaluated. Moreover, these tools rely on KEGG Orthology (KO) and other reference databases for inference but lack clearly defined marker enzymes that can robustly distinguish among the seven known pathways. A key challenge arises from the evolutionary diversity of enzymes: even within a single pathway, phylogenetically distinct enzymes may catalyze equivalent reactions^5^, complicating straightforward rule-based predictions. In addition, the specific marker enzymes required to resolve pathway presence with high confidence have not been systematically defined or curated.

To address these limitations, we first compiled a set of 15 well-characterized chemolithoautotrophic genomes to define pathway-specific marker enzymes and their corresponding KO identifiers (IDs) for all seven CO₂ fixation pathways. Based on this curated marker set, we developed AutoFixMark, a lightweight, rule-based tool that predicts the presence or absence of each pathway from a genome’s KO profile. To evaluate the performance of AutoFixMark, we constructed a separate benchmark dataset comprising 347 genomes from 16 phyla, each manually annotated with literature-based evidence of CO₂ fixation pathway presence. We then compared AutoFixMark’s prediction accuracy with that of two existing tools, METABOLIC and gapseq. Our results indicate that AutoFixMark achieves high sensitivity and specificity across all seven CO₂ fixation pathways. By enabling accurate and interpretable predictions, AutoFixMark facilitates deeper insights into the functional potential of autotrophic microorganisms in diverse environments, including those investigated through metagenomic approaches.

## Methods

### Definition of pathway-specific marker KOs for AutoFixMark

**Fig. 1:**
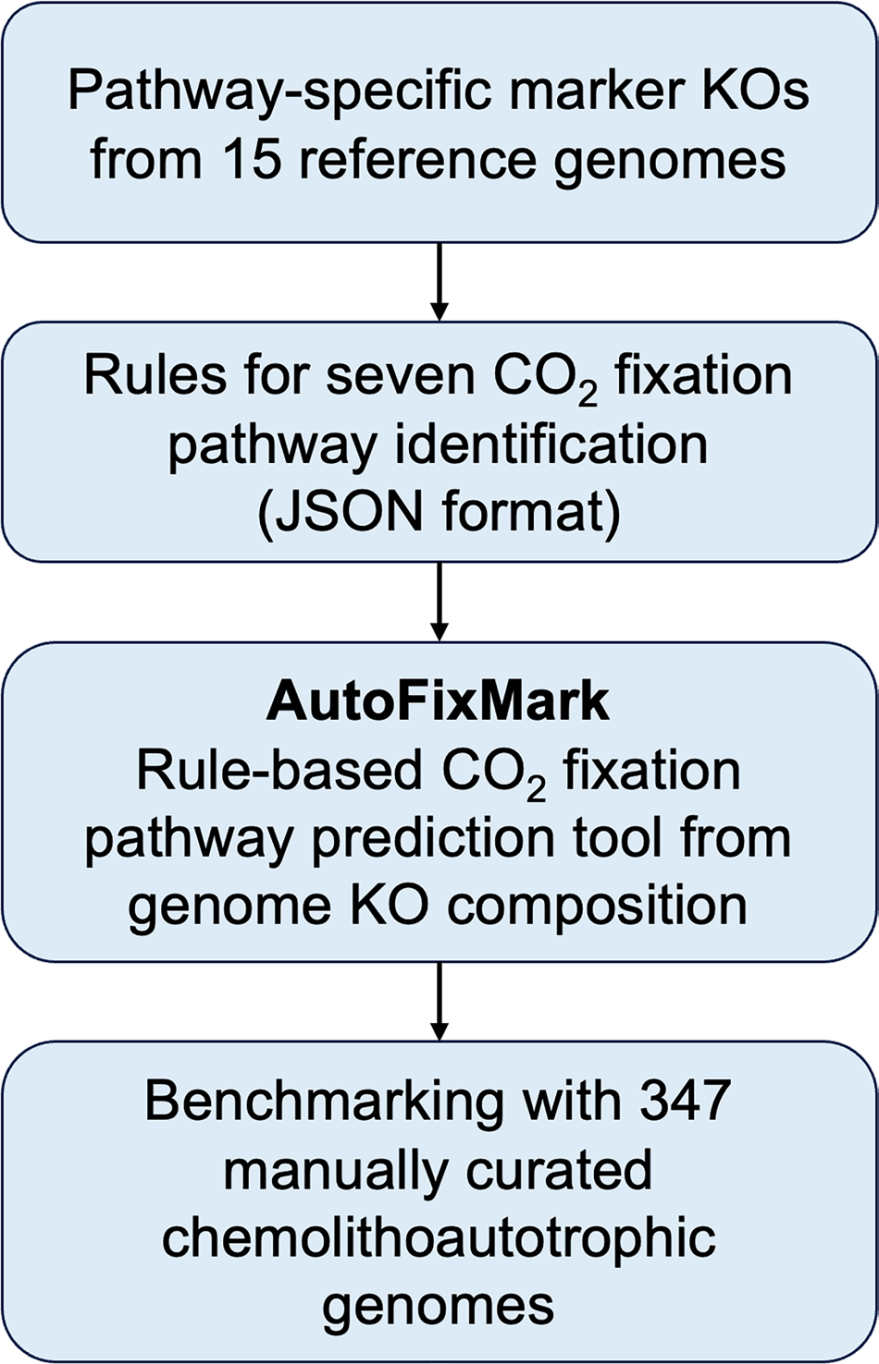
Overview of the study workflow. The diagram summarizes the steps for reference data construction, AutoFixMark development, and tool benchmarking used in this study.

An overview of the study workflow is shown in Fig. 1. To define pathway-specific marker genes for the seven known natural CO₂ fixation pathways, we first reviewed the biochemical architecture of each pathway based on previously published literature, including pathway-focused reviews and primary studies describing enzymatic components indicative of specific pathway types^1,6–9^. While these pathways have been comprehensively described in those reviews, we do not repeat the mechanistic details here. CO₂ fixation pathways often exhibit multiple enzymatic variants that differ in specific reactions, substrates, or cofactors. These variants, observed across diverse microbial taxa, may utilize evolutionarily distinct enzymes that are assigned different KO IDs. We first compiled the genes and corresponding KO IDs involved in each CO₂ fixation pathway using genome information from representative autotrophic microbes. We then selected marker enzymes to identify each pathway. We systematically examined both canonical and non-canonical variants and compiled enzyme-coding genes for each, based on representative strains listed in Table 1. Variants lacking gene-level evidence were excluded from consideration. For each selected marker enzyme, KO IDs were assigned using KofamScan with default parameters^10^ on the RefSeq-derived protein sequences of representative genomes. Hits with scores exceeding the predefined adaptive threshold for each KOfam profile were assigned to the corresponding KO ID. If no hit exceeded the threshold for a given query, the KO ID of the top-scoring hit was assigned instead. When multiple KO IDs exceeded the threshold but clearly included enzymes unrelated to the target pathway, those KO IDs were excluded from further analysis. To account for the functional and evolutionary diversity of enzymes, we implemented a flexible rule-based framework to define the presence of each CO₂ fixation pathway. Three logical rules were used to accommodate pathway-specific features. The “one_of” rule was applied when multiple alternative enzymes (assigned to different KO IDs) could catalyze the same biochemical reaction, indicating that the presence of any one KO is sufficient. The “all_of” rule was used for reactions requiring a multi-subunit enzyme complex, requiring all listed KO IDs to be present. For the rGly pathway, where the essentiality of individual glycine cleavage system subunits remains uncertain, we introduced an “at_least” rule to require a minimum number of subunit KO IDs for prediction. These marker definitions and logical rules are encoded in a machine-readable JSON file, which is used as a core reference by AutoFixMark during prediction. Given a KO profile of a genome, AutoFixMark determines the presence or absence of each of the seven CO₂ fixation pathways based on these predefined rules. The JSON file is publicly available within the AutoFixMark source code repository^11^ and summarized in Table 2.

**Table 1:** List of microbial strains investigated for pathway-specific marker genes identification.

Pathway	Strain name	Genome ID	Reference PubMed IDs
CBB	*Cupriavidus necator* H16	GCF_000009285.1	37002131, 16964242, 23879744, 22961894, 35104625
CBB	*Nitrosospira multiformis* ATCC 25196	GCF_000196355.1	29867847, 18390676
CBB	*Thermodesulfobium acidiphilum* 3127-1	GCF_003057965.1	31451656
rTCA	*Chlorobaculum tepidum* TLS	GCF_000006985.1	20650900, 12093901
rTCA	*Thermovibrio ammonificans* HB-1	GCF_000185805.1	28436819
rTCA	*Hydrogenobacter thermophilus* TK-6	GCF_000010785.1	21740227, 17076668, 18203822, 14731279, 15101981, 15101982, 18757546, 20348262, 16978355
rTCA	*Thermosulfidibacter takaii* ABI70S6	GCF_001547735.1 *1	29420286, 29420287
WL	*Clostridium carboxidivorans* P7	GCF_001038625.1	20885952, 18801467, 16608335, 20877792, 39361653, 18631365
WL	*Methanothermobacter marburgensis* str. Marburg	GCF_000145295.1	21740227, 21559116, 38282645, 21262829, 27458443, 39361653
3HP	*Chloroflexus aurantiacus* J-10-fl	GCF_000018865.1	21714912, 8354269, 19955419, 11948153, 11821399, 19955419, 35889008, 38572988, 9973333, 10.1007/BF00413138
3HP/4HB	*Metallosphaera sedula* DSM 5348	GCF_000016605.1	32218776, 12581213, 18079405, 24532060, 22752162
3HP/4HB	*Nitrosopumilus maritimus* SCM 1	GCF_000018465.1	24843170, 26196861, 25548047, 20421470, 34819551, 34290692
DC/4HB	*Ignicoccus hospitalis* KIN4/I	GCF_000017945.1	18511565, 19000309, 17400748, 12610721, 34161262, 21169482, 34290692
DC/4HB	*Pyrobaculum neutrophilum* V24Sta *3	GCF_000019805.1	20693323
rGly	*Desulfovibrio* sp. G11	GCF_900243745.1	33037220

*1 The rTCA pathway of this strain shares the same enzyme set as the oxidative TCA cycle, making it difficult to distinguish via genome-based analysis; therefore, this pathway was excluded from prediction targets.

*2 Although *Moorella thermoacetica* ATCC 39073 is one of the representative strains for the WL pathway research, its genome has been withdrawn from RefSeq and was thus not included in this study.

*3 No substantial differences in the pathway structure have been reported between *Ignicoccus hospitalis* and this strain; however, *I. hospitalis* has been noted in the literature to lack annotation for certain pathway enzymes, and was therefore included for comparative purposes.

**Table 2:** List of pathway-specific marker enzyme genes.

Pathway	Type	Marker enzyme name	Marker KO IDs
CBB	N.A.	phosphoribulokinase	K00855
CBB	N.A.	ribulose-bisphosphate carboxylase large chain	K01601
CBB	N.A.	ribulose-phosphate 3-epimerase	K01783
rTCA	rTCA-I	ATP-citrate lyase	K15230 and K15231
rTCA	rTCA-II	citryl-CoA synthetase	K15233 and K15232
rTCA	rTCA-II	citryl-CoA lyase	K15234
WL	WL-I	anaerobic carbon-monoxide dehydrogenase catalytic subunit	K00198
WL	WL-I	acetyl-CoA synthase	K14138
WL	WL-II	CODH/ACS complex subunit alpha + epsilon	K00192 and K00195
WL	WL-II	CODH/ACS complex subunit beta + delta + gamma	K00193, K00194, and K00197
3HP	N.A.	malonyl-CoA reductase / 3-hydroxypropionate dehydrogenase (NADP +)	K14468
3HP	N.A.	succinyl-CoA-L-malate CoA-transferase	K14471 and K14472
3HP/4HB	3HP/4HB-I	malonyl-CoA reductase	K15017
3HP/4HB	3HP/4HB-I	3-hydroxypropionate dehydrogenase (NADP +)	K15039
3HP/4HB	3HP/4HB-I	4-hydroxybutanoyl-CoA dehydratase	K14534
3HP/4HB	3HP/4HB-II	acetyl-CoA carboxylase	K18603, K18604, and K18605
3HP/4HB	3HP/4HB-II	malonic semialdehyde reductase	K18602
3HP/4HB	3HP/4HB-II	4-hydroxybutanoyl-CoA dehydratase	K14534
DC/4HB	N.A.	phosphoenolpyruvate carboxylase	K01595
DC/4HB	N.A.	fumarate hydratase	K01676 or K01677 or K01678
DC/4HB	N.A.	4-hydroxybutyrate-CoA ligase	K18861 or K14467
DC/4HB	N.A.	4-hydroxybutanoyl-CoA dehydratase	K14534
rGly	N.A.	glycine cleavage system	at least three of K00283, K00282, K02437, and K00605
rGly	N.A.	glycine reductase	at least five of K10671, K10672, K00384, K21577, K21576, K03671, and K10670

### Marker enzyme genes for the Calvin–Benson–Bassham (CBB) cycle

For the Calvin–Benson–Bassham (CBB) cycle, we selected three marker enzyme genes: ribulose-1,5-bisphosphate carboxylase/oxygenase (RubisCO) large subunit [K01601] and phosphoribulokinase (PRK) [K00855], which synthesizes ribulose-1,5-bisphosphate^12^, and ribulose-phosphate 3-epimerase (RPE) [K01783]. Although RubisCO typically functions as a hetero-oligomer that includes a small subunit [K01602], some organisms are known to possess functional CBB cycles in the absence of the small subunit^13^; therefore, K01602 was not included as a required marker. Both PRK and the RubisCO large subunit have also been identified in certain archaeal species^14^, where they are hypothesized to function as part of the reductive hexulose-phosphate (RHP) pathway. The pathway was considered as a primitive prototype of the CBB cycle^15^. To distinguish between the CBB cycle and the RHP pathway, we included RPE, one of the enzymes that regenerates ribulose 5-phosphate.

### Marker enzyme genes for the reductive tricarboxylic acid (rTCA) cycle

The reductive tricarboxylic acid (rTCA) cycle can be broadly divided into two mechanistic types: an asymmetric type (rTCA-I) and a symmetric type (rTCA-II), each characterized by distinct enzymatic steps^16^. In AutoFixMark, we considered the presence of either type to be indicative of the rTCA pathway. For rTCA-I, we selected ATP citrate lyase [K15230 and K15231] as the marker enzyme. For rTCA-II, we used a combination of citrate-CoA ligase [K15233 and K15232] and citryl-CoA lyase [K15234] as marker enzymes^7^. It is important to note that in some organisms, the conversion of citrate to acetyl-CoA is carried out by citrate synthase (an enzyme shared with the oxidative TCA cycle) resulting in a bidirectional or reversible TCA cycle^17,18^. In such cases, the genome lacks unique marker enzymes that distinguish rTCA from oxidative TCA, and therefore pathway prediction based on marker genes is not feasible. Such discrimination is beyond the scope of the current study and is left for future work.

### Marker enzyme genes for the Wood–Ljungdahl (WL) pathway

The Wood–Ljungdahl (WL) pathway exists in two phylogenetically distinct forms: the bacterial type (WL-I), represented by acetogenic bacteria, and the archaeal type (WL-II), represented by methanogenic archaea^7^. In both cases, the key marker enzymes are anaerobic carbon monoxide dehydrogenase (CODH) and CO-methylating acetyl-CoA synthase (ACS)^7^, although the assigned KO IDs differ by lineage. For WL-I, the marker KO IDs are CODH [K00198] and ACS [K14138]. For WL-II, the corresponding marker set includes CODH [K00192, K00195] and ACS [K00193, K00194, K00197]. Importantly, the WL pathway is reversible, and even the complete presence of marker enzymes and associated genes does not necessarily indicate the capability for autotrophic growth^19^. In addition, hybrid variants that combine bacterial- and archaeal-type CODH/ACS subunits have been reported^20^, including our previous study^5^. These cases may require the development of separate classification rules in future versions of the tool.

### Marker enzyme genes for the 3-hydroxypropionate (3HP) bicycle

The key enzyme for the 3-hydroxypropionate (3HP) bicycle is malonyl-CoA reductase/3-hydroxypropionate dehydrogenase [K14468], which catalyzes the formation of 3-hydroxypropionate from malonyl-CoA^21^. To differentiate this pathway from the 3HP/4HB cycle, we also included succinyl-CoA:L-malate CoA-transferase [K14471 and K14472] as a marker enzyme, as it is absent in the 3HP/4HB pathway.

### Marker enzyme genes for the 3-hydroxypropionate/4-hydroxybutyrate (3HP/4HB) cycle

As with the 3HP bicycle, the first marker enzyme for the 3-hydroxypropionate/4-hydroxybutyrate (3HP/4HB) cycle is malonyl-CoA reductase [K15017], though it is associated with a different KO ID than that used for the 3HP bicycle. To distinguish this pathway from the DC/4HB cycle, we also included 3-hydroxypropionate dehydrogenase / malonic semialdehyde reductase [K15039], which catalyzes the subsequent step.

### Marker enzyme genes for the dicarboxylate/4-hydroxybutyrate (DC/4HB) cycle

The dicarboxylate/4-hydroxybutyrate (DC/4HB) cycle shares partial routes with both the rTCA cycle and the 3HP/4HB cycle^6^, making it difficult to detect without a broader set of marker enzymes. We selected 4-hydroxybutyrate-CoA ligase [K18861 or K14467] and 4-hydroxybutanoyl-CoA dehydratase [K14534] as marker enzymes in the 4HB part. To differentiate this pathway from the 3HP/4HB cycle, we also included phosphoenolpyruvate carboxylase [K01595] and fumarate hydratase [K01676, K01677, or K01678] as marker enzymes. However, we note that certain strains assigned to the 3HP/4HB cycle possess most of the gene set for the DC/4HB cycle, making differentiation difficult based solely on gene presence. Therefore, further evidence from metabolic studies or experimental validation may be required to distinguish the functional use of these pathways.

### Marker enzyme genes for the reductive glycine (rGly) pathway

The reductive glycine (rGly) pathway is a recently discovered CO₂ fixation pathway, first described in 2020^22^, and its phylogenetic distribution remains largely unknown. According to the original study, the glycine reductase complex in which comprising seven subunits [K10671, K10672, K00384, K21577, K21576, K03671, K10670], was identified as a unique and characteristic feature of the pathway. In addition, the glycine cleavage system in which involving four enzymes [K00283, K00282, K02437, K00605], plays a central role in glycine metabolism. Therefore, both complexes were designated as marker enzyme sets. Following the detection criteria described in the original publication, we considered the presence of at least 5 out of 7 glycine reductase subunits and at least 3 out of 4 glycine cleavage system components as markers for the pathway. The conversion of glycine to pyruvate in this pathway can proceed either via serine or via acetyl-CoA. As the latter route was proposed to be dominant under autotrophic growth conditions, we selected enzymes specific to the acetyl-CoA–mediated route as additional markers.

### Benchmark genome dataset construction

To construct a benchmark dataset for CO₂ fixation pathway prediction, we first conducted a PubMed literature search using the keyword “CO₂ fixation” and similar words to identify candidate microbial species. Retrieved literatures were manually screened to extract microbial species names and their associations with CO₂ fixation activity. Since our focus was on chemolithoautotrophs, photosynthetic organisms were excluded. However, a subset of phototrophic species harboring the 3HP bicycle was retained to ensure representation of this pathway^21^. For each identified species, we searched for available genome sequences in the NCBI RefSeq and GenBank databases^23^. Genome selection followed a prioritized scheme: (i) type strains in RefSeq, (ii) non-type strains in RefSeq, and (iii) strains in GenBank. This process yielded an initial set of 460 genomes. We subsequently excluded organisms that exhibited only anaplerotic CO₂ fixation or lacked clear evidence of autotrophic growth^24^. The final curated dataset comprised 347 genomes representing confirmed chemolithoautotrophs^25^. Pathway presence for each species was manually identified through literature review. The level of supporting evidence varied across species and included biochemical enzyme assays, gene expression data, and inferences based on partial gene sets. In cases where direct evidence was unavailable, pathway presence was inferred based on closely related species with experimentally validated pathway presence.

### Benchmarking of pathway prediction tools

We evaluated the performance of three tools (i.e., AutoFixMark, METABOLIC, and gapseq) in predicting CO₂ fixation pathways across the 347 benchmark genomes. To eliminate differences arising from protein-coding gene prediction, we used the RefSeq-annotated protein sequences from each genome in all three tools. For AutoFixMark, KO IDs were assigned to each protein sequence using the KofamScan with default parameters. Hits with scores exceeding the predefined adaptive threshold for each KOfam profile were assigned to the corresponding KO ID. If no hit exceeded the threshold for a given query, the KO ID of the top-scoring hit was assigned instead. METABOLIC version 4.0 was performed using default parameters. gapseq version 1.4 was executed with a custom pathway list (LWP-GS, CALVIN-PWY, P23-PWY, P42-PWY, PWY-5392, CODH-PWY, PWY-778, PWY-8303, PWY-5743, and PWY-5789). For each genome, the presence or absence of each pathway was predicted by all three tools and summarized into a binary presence/absence matrix. The predictions were then compared against the manually curated reference dataset to evaluate accuracy. For each pathway, we computed precision, recall, and F1 statistics based on the number of true positive, false positive, and false negative predictions across all genomes.

## Data Records

The rule-based definitions of the marker KO ID combinations for the seven CO₂ fixation pathways are provided in a JSON file (kegg_key_enzymes.json), which is publicly available in the definitions folder of the AutoFixMark GitHub repository^11^. The rule-based pathway prediction tool AutoFixMark is implemented in Python and can be run as a standalone script without requiring external library dependencies. AutoFixMark Python script (predict_pathways.py) is distributed under the MIT license and is available in the app folder of the repository. Since AutoFixMark requires a genome-derived KO list as input, a format conversion Python script (kofamscan_parser.py) is also provided in the app folder to convert the TSV output from the KO assignment software KofamScan^10^ into the simple KO list.

The benchmark dataset consists of 347 genomes representing confirmed CO₂-fixing microbes and is available via Zenodo^25^. The dataset includes: (i) an Excel file containing the genome ID (INSDC GCA/GCF ID), species name, phylum name, CO₂ fixation pathway, rationale for pathway identification, and references (PubMed ID or DOI) for both pathway identification and experimental evidence of CO₂ fixation; and (ii) protein sequence FASTA files for the 347 genomes, with each file containing all protein sequences of a genome. The Excel file also lists 12 reference genomes of CO₂ fixation pathways; however, these were not included in the benchmark analysis and therefore their protein sequence FASTA files are not provided.

## Data Overview

### The pathway-specific marker KO IDs and AutoFixMark tool description

In AutoFixMark version 1, pathway-specific KO IDs were defined for each of the seven CO₂ fixation pathways as follows: the CBB cycle: 3 KOs; the rTCA cycle: 5 KOs; the WL pathway: 7 KOs; the 3HP bicycle: 3 KOs; the 3HP/4HB cycle: 7 KOs; the DC/4HB cycle: 7 KOs; and the rGly pathway: 11 KOs. The rule-based definitions of these KO IDs combinations for each pathway are implemented in a JSON format file and are publicly available^11^. AutoFixMark is implemented in Python and can be installed as a standalone script without the need for external library dependencies. AutoFixMark requires a genome-derived KO list as input. KofamScan TSV outputs can be converted into the appropriate KO list format using our conversion Python program.

### Benchmark genome dataset description

**Fig. 2:**
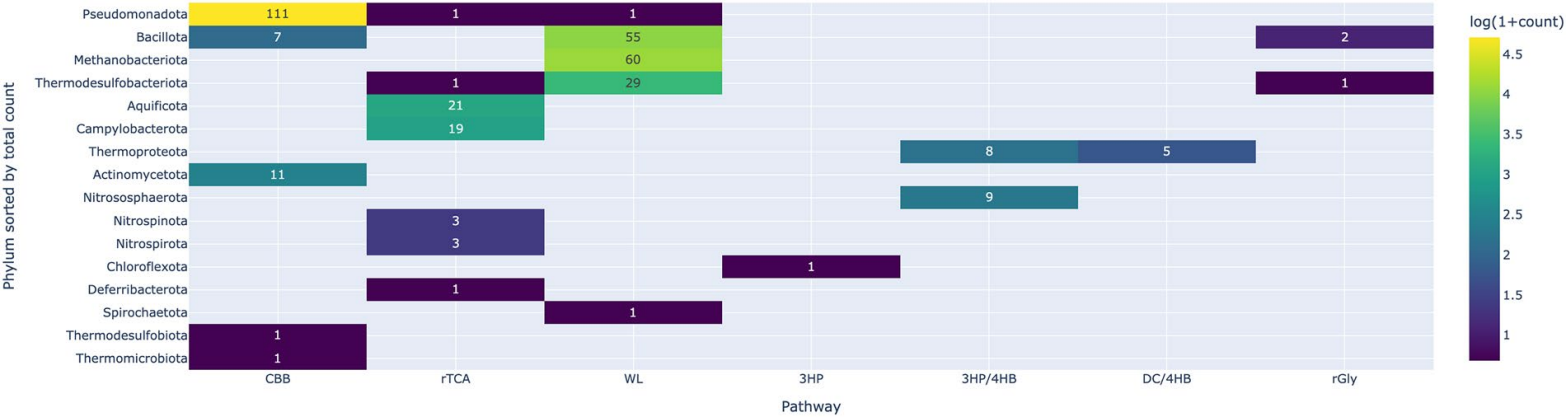
Heatmap of the phylogenetic distribution of seven CO_2_ fixation pathways. The figure summarizes the phylum-level distribution of the seven CO₂ fixation pathways across the 347-genome benchmark dataset. Colors indicate the number of genomes possessing each pathway.

The benchmark genome dataset comprises 347 genomes representing confirmed CO₂-fixing microbes^25^. These genomes span 16 bacterial and archaeal phyla. The number of genomes predicted to possess each of the seven CO₂ fixation pathways is summarized in Fig. 2. Notably, due to the limited number of sequenced genomes and available pathway annotations for certain pathways, particularly the 3HP bicycle, the DC/4HB cycle, and the rGly pathway, the benchmark performance results for these pathways should be interpreted with caution. The total count of 352 pathways across 347 genomes reflects the presence of strains harboring multiple CO₂ fixation pathways. Specifically, three strains in the phylum *Bacillota* possess both the CBB and WL pathways, while two strains in the phylum *Bacillota* exhibit a combination of the WL and rGly pathways.

## Technical Validation

**Fig. 3:**
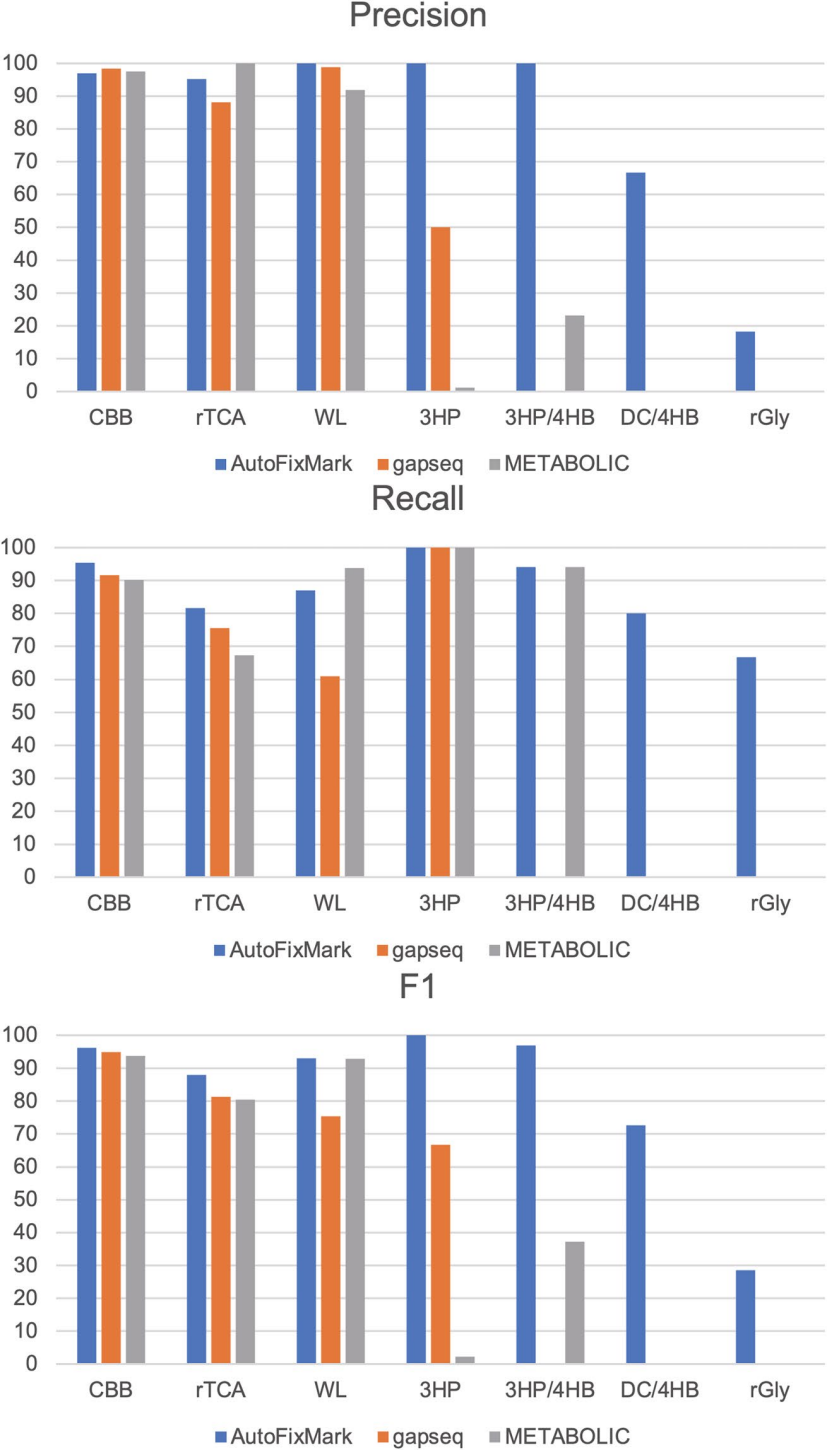
Benchmark results of three tools. Precision, recall, and F1 statistics were calculated using the 347-genome benchmark dataset. For pathways not supported by a given tool, precision, recall, and F1 scores were set to 0.

We evaluated the predictive performance of AutoFixMark against two existing tools, METABOLIC and gapseq, using a benchmark dataset of 347 manually curated chemolithoautotrophic genomes. METABOLIC predicts metabolic pathways by identifying homologous proteins of marker genes across KEGG modules or custom-defined gene collections through HMMER-based searches against curated HMM profiles and assigning KO IDs based on sequence similarity^3^. gapseq annotates metabolic genes through BLAST- and HMM-based similarity searches and gapseq’s pathway inference relies on gene evidence scoring, network-consistency checks, and gap-filling heuristics to account for incomplete genomes or missing annotations^4^. In contrast, AutoFixMark takes as input the KO composition of a genome’s protein set, typically generated using KofamScan, and predicts the presence of the seven known CO₂ fixation pathways by evaluating explicitly defined combinations of pathway-specific marker KO IDs. The precision, recall, and F1 scores for each of the seven known CO₂ fixation pathways are summarized in Fig. 3. For the well-characterized pathways (i.e., the CBB cycle, the rTCA cycle, and the WL pathway), all three tools achieved high prediction accuracy. For the 3HP bicycle, pathway annotation remains extremely limited; only one genome (*Roseiflexus castenholzii*) in the benchmark set is annotated with this pathway, precluding meaningful precision or recall analysis at this stage. For the less-studied or more recently discovered pathways (i.e., 3HP/4HB, DC/4HB, and rGly pathway), AutoFixMark outperformed existing tools, some of which do not include these pathways in their prediction scope. In particular, AutoFixMark was able to make predictions for pathways entirely unsupported by METABOLIC or gapseq. The DC/4HB pathway was predicted exclusively by AutoFixMark, as the other two tools do not support prediction of this pathway. In addition, METABOLIC lacks a prediction function for the rGly pathway. The lower prediction accuracy of METABOLIC and gapseq for the 3HP, 3HP/4HB, and rGly pathways (in the case of gapseq) is likely due to insufficient marker gene definitions in their reference databases. The rGly pathway, first described in 2020^22^, remains poorly characterized in terms of its taxonomic distribution. While AutoFixMark showed relatively low precision for rGly, it is possible that these predictions reflect true but as-yet unconfirmed pathway presence. Further experimental validation and the accumulation of supporting literature are needed to clarify the biological relevance of these predictions.

AutoFixMark enables accurate and interpretable prediction of CO₂ fixation potential from microbial genomes, including metagenome-assembled genomes. Its high accuracy primarily stems from the carefully curated, pathway-specific marker gene sets and the rule-based framework that allows precise discrimination among CO₂ fixation pathways that share homologous enzymes. By facilitating the identification of autotrophic microbes in carbon-limited environments, it contributes to a deeper understanding of microbial carbon metabolism and the ecological roles of autotrophs across diverse ecosystems. Importantly, AutoFixMark is intended as a first-pass screening tool to identify organisms with genetic potential for autotrophic CO₂ fixation, not to infer active metabolism or growth phenotype. The presence of marker genes alone does not guarantee pathway expression or functional activity, especially given that many CO₂ fixation pathways are biochemically reversible and may operate in either direction depending on the organism’s metabolic and energetic context. Accordingly, AutoFixMark does not infer flux directionality. As new studies continue to uncover alternative enzymes or novel variants of known pathways, we plan to update and expand the marker gene definitions in AutoFixMark to reflect the evolving understanding of microbial autotrophy.

## Data Availability

The rule-based definitions of KO marker combinations and the AutoFixMark Python program are available at the AutoFixMark GitHub repository (https://github.com/h-mori/AutoFixMark). The benchmark genome dataset is available from Zenodo (10.5281/zenodo.16956127).

## Code availability

The AutoFixMark program and the marker gene definition JSON file are publicly available within the AutoFixMark source code repository (https://github.com/h-mori/AutoFixMark).
